# Supplementary material for: Ethiopian antimicrobial consumption trends in human health sector: A surveillance report 2020–2022
Source: PLoS One. 2025 Feb 28;20(2):e0319295. doi: 10.1371/journal.pone.0319295 (PMC11870371; doi:10.1371/journal.pone.0319295)
Supplement: S1. File — (DOCX) [file pone.0319295.s001.docx]

**S1 file: Antimicrobials excluded from AMC analysis**

The following items of fixed dose combination antimicrobials, which were consumed in Ethiopia for the treatment of HIV/AIDS and Mycobacterium tuberculosis and excluded in the analysis since DDD value was not available:

1. Rifampicin 75mg/Isoniazid 50mg/Pyrazinamide 150mg/Ethambutol hydrochloride 50mg
2. Rifapentine 300mg + INH 300mg
3. Rifapentine 300mg + INH 300mg
4. Rifapentine 300mg/ Isoniazed 300mg
5. Rifapentine/Isoniazid 300/300 mg Film-Coated Tablet
6. Tenofovir 300mg + Lamivudine 300mg + Dolutegravir 50mg
7. Tenofovir 300mg + Lamivudine 300mg + Efavirenz 400mg
