# Supplementary material for: Ethiopian antimicrobial consumption trends in human health sector: A surveillance report 2020–2022
Source: PLoS One. 2025 Feb 28;20(2):e0319295. doi: 10.1371/journal.pone.0319295 (PMC11870371; doi:10.1371/journal.pone.0319295)
Supplement: Attachment — Submitted filename: PLOS One review.pdf [file pone.0319295.s003.pdf]

PLOS One review

Thank you for inviting me to review this paper: “Antimicrobial consumption trend in Ethiopia: A surveillance report 2020-2022”
An interesting study which gives the overall trends of AMC in Ethiopia over 3 years. The paper is well written with regards to language and the methods used give scientifically valid observations. However, the discussion can be improved.

Following are my detailed observations:

Title:
Would be better if “trends” is used instead of ‘trend’

Abstract:
Line 30: ‘respectively’ can be deleted as it is superfluous
Line 29: “…were in the Access group” – which Access group are the authors referring to as they have used two AWaRe classifications

Introduction:
Line 70: It would be better to state that the study is limited to AMC of humans. It is mentioned at the end as a limitation but if the study is specifically to look at AMC in humans, it will not be a limitation

Adding a brief description of how Ethiopia obtains antimicrobials would be helpful to understand the context.

Methods:
It is not clear from the manuscript and Figure 1 whether the data that was used for the analysis is only those that were used for human consumption as antimicrobials are also used for animals.
If this was so, how the amounts for human consumption was extracted from the point of data collection must be stated in the manuscript under methodology and figure 1 should be updated.

Line 76: “Specific data collection points” – are these selected points or all entry points of antimicrobials into Ethiopia?

Line 91: ATC/DDD 2023 index – citation needed. Once the citation is added, please make sure that those that follow are renumbered and cited appropriately.

Line 103: “abstraction” – shouldn’t this be “extraction”?

Lines 127 & 128: Are the WHO and Ethiopian AWaRe classifications different? If so, what are the key differences? The rationale for having a separate AWaRe classification from that of the WHO in Ethiopia should be stated and reference for Ethiopian AWaRe classification should be cited.

Results
Table 1: WPP* - what this means should be stated as a footnote to the table

Line 168: If ‘Whereas’ is used, the two sentences should be combined to read as one.

Table 4: Why is there a * with Antibacterials (Antibacterials*) as these are both imported and locally manufactured?

Lines 184 & 185: “Agents against amoebiasis and …” should be corrected as “Consumption of Agents against amoebiasis and….” (these agents are also used as antibacterials, probably the reason for widespread use)

Lines 197 & 198: The discrepancy between these two AWaRe classifications should be discussed in the methods

Line 206: procaine benzyl penicillin is not among DU75 for 2022 but was there for 2020 and 2021. The sentence should be corrected to reflect this.

Table 7 What are the units for antimicrobial consumption? It appears to be the percentage of the total consumption, it should be stated to improve clarity

Discussion:
There are several trends identified in the results, however, the discussion based on these can be improved.

Trend 1. Line 148: Increased AMC from ’20 to ’21 but a decrease in ‘22

This is discussed in lines 233-241. The authors discuss this in relation to Uganda and Tanzania, but it is unclear why these countries have been selected for comparison.
It is also unclear how COVID pandemic affected these countries differently as regards to AMC as discussed by the authors. The effects of COVID were highest in 2020 and 2021 in most countries and with lockdowns, a reduction in AMC can be expected. However, Ethiopia shows a reduction of AMC in 2022 when the effects of COVID was decreasing in most countries. If the antimicrobial stewardship programmes are not well established in Ethiopia, Uganda and Tanzania and if the COVID pandemic affected the countries in a similar manner, the findings cannot be explained as the authors have done.

Lines 184-185: Significant consumption of P01AB – ie agents against amoebiasis and protozoal diseases – is this because of metronidazole as it is also used for anaerobic bacterial infections or was there an increase in amoebiasis and protozoal infections?

Lines 251 – 254: It does not appear from the results that there has been a disruption of antimicrobial supply to Ethiopia during the COVID pandemic. The statements in lines 251-254 must be supported with appropriate references.

Line 262: Reference to Ethiopian standard treatment guidelines should be provided

Line 262 – “In addition” – to be deleted

Lines 285-287: What does this mean? Are there antimicrobials in Ethiopia that are outside the WHO AWaRe classification? If so, what are they?

Line 295 – 296: “sulphamethoxazole + trimethoprim” – this is ‘co-cotrimoxazole’ and can be written as such or ‘trimethoprim/sulfamethoxazole’

Lines 304-305: Generic medicinal drug names are not proper nouns, l/c should be used in all places unless it is at the beginning of a sentence.

Trend 2.
Lines 186-187: increased use of J01CA – extended spectrum penicillins
Line: 188-189 – increased 3rd generation cephalosporins
– the significance of these for AMR should be discussed.

Trend 3:
Lines 217-221: Antimalarials – the possible reasons for a significant decrease in artemether and artesunate consumption should be discussed -e.g. changes in policy/treatment guidelines

Strengths and Limitations
Lines 322 – 323: This can be stated in the methodology and will not be a limitation then.

I am also sharing the link to a paper on AMC done by my group for your information, you can see how some quality indices from ESAC we have used, which is useful to see the trends in AMC. https://doi.org/10.1371/journal.pone.0257424
**Please note that you do NOT have to cite this paper unless you feel that it would help yours in anyway.
