# Supplementary material for: Ethiopian antimicrobial consumption trends in human health sector: A surveillance report 2020–2022
Source: PLoS One. 2025 Feb 28;20(2):e0319295. doi: 10.1371/journal.pone.0319295 (PMC11870371; doi:10.1371/journal.pone.0319295)
Supplement: Attachment — Submitted filename: Response to reviewers.docx [file pone.0319295.s002.docx]

**Date: November 2, 2024**

**Point by point response**

**Dear Editor and Reviewers**

We are very grateful for the comments provided for our article entitled “**Antimicrobial consumption trend in Ethiopia: A surveillance report 2020-2022”** this manuscript.

We revised the manuscript as per the valuable comments and summarized our response to the comments as below along with the an unmarked version of our revised manuscript without tracked changes as *“Manuscript”* and a marked-up copy of our manuscript that highlights changes made to the original version as “*Revised Manuscript with Track Changes*”.

| **Comment** | **Response** |
| --- | --- |
| Please ensure that your manuscript meets PLOS ONE's style requirements, including those for file naming. | Thank you so much. We revised the manuscript as per PLOS ONE requirements (see the marked manuscript copy for your quick reference) |
| Please ensure that you refer to Figure xxxxx in your text as, if accepted, production will need this reference to link the reader to the figure. | Thank you so much and we hereby confirm the figure caption and citation are as per the PLOS ONE author’s guideline. |
| Please review your reference list to ensure that it is complete and correct. If you have cited papers that have been retracted, please include the rationale for doing so in the manuscript text, or remove these references and replace them with relevant current references. Any changes to the reference list should be mentioned in the rebuttal letter that accompanies your revised manuscript. If you need to cite a retracted article, indicate the article’s retracted status in the References list and also include a citation and full reference for the retraction notice. | The reference list is reviewed and hereby confirm the completeness and correctness of the list. |
| Reviewer #2: The work is insightful and the article reported what was on ground for the period of the surveillance. The authors assumed that total quantity of Antimicrobials of interest imported and all manufactured locally equals the consumed Antimicrobials within the period. They didn't make exceptions of those that could have been exported informally and those expired. This estimate quantity should be factored in determining the total AMC. In that regard authors should include it under study limitations. | Indeed thank you so much for the valuable comment. Now, we included the same as a limitation on page 20 line number 324-326 as “Some antimicrobials may have been informally exported (i.e., without official records) or expired before use. As a result, not all antimicrobials included in the total supply were necessarily consumed in Ethiopia.” |
