# Supplementary material for: Ethiopian antimicrobial consumption trends in human health sector: A surveillance report 2020–2022
Source: PLoS One. 2025 Feb 28;20(2):e0319295. doi: 10.1371/journal.pone.0319295 (PMC11870371; doi:10.1371/journal.pone.0319295)
Supplement: Attachment — Submitted filename: Point by point response Jan 28 2025.docx [file pone.0319295.s006.docx]

**Date: January 28, 2025**

**Point by point response**

**Dear Editor and Reviewers**

We are very grateful for the comments provided for our article entitled “**Antimicrobial consumption trend in Ethiopia: A surveillance report 2020-2022”** with manuscript ID: **PONE-D-24-39610R3** for the second time.

We revised the manuscript as per the valuable comments and summarized our response to the comments as below along with the an unmarked version of our revised manuscript without tracked changes as *“Manuscript”* and a marked-up copy of our manuscript that highlights changes made to the original version as “*Revised Manuscript with Track Changes*”.

| **Comment** | **Response** |
| --- | --- |
| **Editor’s and Reviewer #4 comments** |  |
| **Discussion** |  |
| I thank the authors for providing possible explanations for the increase in antibiotic consumption. However, in my previous review, I referred to antimalarial drugs, not antibiotics. I would like the authors to provide explanations for the trends in antimalarial drug use. If the manuscript is revised accordingly, I propose that the editors accept it for publication without requiring further review from me. | Thank you for this valuable comment once again and we are very sorry for the misunderstanding of your query. Now, we tried to include a possible explanation why antimalarial consumption was increased in the year 2021 in Ethiopia (Refer line 310-317). |
