# Supplementary material for: Ethiopian antimicrobial consumption trends in human health sector: A surveillance report 2020–2022
Source: PLoS One. 2025 Feb 28;20(2):e0319295. doi: 10.1371/journal.pone.0319295 (PMC11870371; doi:10.1371/journal.pone.0319295)
Supplement: Attachment — Submitted filename: Point by point response Jan 16 2025.docx [file pone.0319295.s005.docx]

**Date: December 25, 2024**

**Point by point response**

**Dear Editor and Reviewers**

We are very grateful for the comments provided for our article entitled “**Antimicrobial consumption trend in Ethiopia: A surveillance report 2020-2022”** with manuscript ID: **PONE-D-24-39610R1**.

We revised the manuscript as per the valuable comments and summarized our response to the comments as below along with the an unmarked version of our revised manuscript without tracked changes as *“Manuscript”* and a marked-up copy of our manuscript that highlights changes made to the original version as “*Revised Manuscript with Track Changes*”.

| **Comment** | **Response** |
| --- | --- |
| **Editor’s and Reviewer #5 comments** |  |
| **Title and abstract** |  |
| Update the title to use “trends” instead of “trend” to reflect the nature of the analysis. | Thank you for this suggestion. We have updated the title to “Ethiopian antimicrobial consumption trends in human health sector: A Surveillance Report 2020-2022”also to better reflect the scope of the AMC. |
| Clarify the **“Access group”** reference in the abstract, ensuring it specifies whether it refers to the Ethiopian AWaRe or WHO AwaRe classification. | Thank you for highlighting this point. We have revised the abstract to explicitly indicate that the “Access group” refers to the Ethiopian AwaRe classification (line 39-40). |
| **Introduction** |  |
| Clearly state that the study focuses on **human antimicrobial consumption** (AMC). | Thank you for pointing out this clarification. We have added a statement to the **Introduction** explicitly indicating that the study exclusively focuses on human AMC (Line 70-71). |
| Provide an explanation of Ethiopia's **process for obtaining antimicrobials**, including procurement and distribution. | Thank you for this valuable suggestion. We have expanded the introduction to include a description of Ethiopia's process for obtaining antimicrobials, including the procurement and distribution mechanisms from Lines 70-73. |
| **Methods** |  |
| Specify if the "specific data collection points" are selected or all points of entry. | Thank you so much for the comment. Now we indicated the same in the manuscript as we included all the ports of entry in line number 81. |
| Detail how **human-specific data** were extracted to ensure clarity regarding the exclusion of animal antimicrobial consumption. | Thank you for this suggestion. Our data sources were local human medicine manufactures the produce antimicrobials and the imported antimicrobials from the human medicine regulatory authority EFDA that controls all the ports of entries (Line 87-92). A different regulatory body called Ethiopian Agricultural Authority regulates medicines including antimicrobials for animal sector manufacturing and import. |
| Use "extraction" instead of "abstraction" to avoid confusion. | Thank you and now changed it to extraction (Line number 109. |
| Clarify the differences between the **Ethiopian AWaRe classification** and the **WHO AWaRe classification** and provide references to support this comparison. | Thank you for the valuable comments that will definitely create clarification for the AMC trend analysis part as per AWaRe. Some of the antimicrobials categorized under access group and watch group were classified as watch and reserve group in the Ethiopian AWaRe classification, and vice versa. The Ethiopian AWaRe categorization only categorized antimicrobials included in the Ethiopian Essential medicine list which in were in total 33 (Access: 16, Watch: 10 and Reserve: 07). Whereas in the WHO AWaRe a total of (Access: 141; Watch: 87 and Reserve: 29). Example, Vancomycin was categorized as Reserve in Ethiopia while watch in WHO; Clarithromycin, Azithromycin and Norfloxacin are Watch in WHO and Access in Ethiopia. Erythromycin is not categorized in Ethiopian AWaRe, but in WHO it is Watch. Now the quick reference for the readers, we included the link (Line 134-136). |
| Provide a citation for the ATC/DDD 2023 index and ensure proper numbering for all references. | Thank you and now we included the same as in text citation and put it in the reference section with proper numbering. |
| State how antimicrobials that lack ATC codes and DDDs were handled and consider tabulating this information in a supplementary table. | Thank you for the comment. Now, we included the same as a supplementary and indicated it as S1 file in the body of the Manuscript (Line 98-99). |
| Address discrepancies in antimicrobial categories and clarify missing data for D01BA (2022) and A07AA (entire study period). | The comment is valid and now to make it clear, we included in the revised manuscript Table 1, why D01BA (2022) and A07AA (entire study period) were not included in the report as a footnote (Line 144-146). |
| Clarify the meaning of WPP* with a footnote in Table 1. | Thank you and now included the full word as footnote. |
| **Results** |  |
| Merge two sentences related to “Whereas” (Line 168) into one for clarity. | Thank you and now we merged the two sentences. |
| Ensure consistency in reporting the units for antimicrobial consumption (e.g., DID, tons, percentage) and clearly label units in tables (e.g., Table 7). | Thank you and now we included DID in table 7 in addition to percentage. However, the reason why we excluded tones in the rest of tables is because WHO doesn’t considered it as a standard metrics for human AMC. |
| Address missing or excluded antimicrobials from the ATC classification and explain the proportion of total consumption they represent. | Thank you for the comment. Unfortunately, we can’t estimate the proportion of the excluded antimicrobials (i.e fixed dose combinations for TB and HIV treatment). GLASS also excluded these products in the analysis. |
| Justify the removal of D01BA in 2022 and the absence of A07AA in the study period. | Thank you and we have already addressed this in the method section. Refer Line number 144-146. |
| **Discussion** |  |
| Discuss observed trends in AMC, particularly the increase from 2020 to 2021 and the decrease in 2022. | Thank you for this valuable comment. We have expanded the Discussion section to analyze the observed trends in AMC (Refer line 242-247). |
| Compare Ethiopia’s trends with other countries like **Uganda and Tanzania**, emphasizing differences in AMC trends due to COVID-19. | Thank you for this suggestion. However, we couldn’t able to get a possible published evidence why such difference happened during COVID-19. |
| Address the significant usage of metronidazole (P01AB) for amoebiasis, noting that it is also used for anaerobic bacterial infections. | Thank you. Now we reflected the same in the discussion section. |
| Explain why certain antimicrobial agents, like procaine benzyl penicillin, appeared in 2020 and 2021 but not in 2022. | Thank you for the valuable comments, and now re-written the sentence to reflect that procaine benzyl penicillin was not in DU75 for the year 2022 |
| Highlight the implications of increased use of extended-spectrum penicillins (J01CA) and 3rd-generation cephalosporins in the context of antimicrobial resistance (AMR). | Thank you for this important comment. We have expanded the Discussion section to address the increased use of extended-spectrum penicillins and third-generation cephalosporins and their implications for AMR. |
| Discuss potential reasons for the decrease in consumption of antimalarials (e.g., policy or treatment guideline changes). | Thank you for the suggestion. Unfortunately, because of lack of documented evidence why the consumption was increased in 2021 and decreased in 2022, we are unable to justify it. In contrary, there is even a malaria outbreak this year and with expected significant increment of consumption. |
| Clarify statements about disruptions in Ethiopia's antimicrobial supply during COVID-19 and support these claims with evidence. |  |
| **Reviewer #3** |  |
| This study is commendable in that it makes every effort possible to clarify the situation of AMC in Ethiopia based on the information available. On the other hand, since readers in other countries do not have a good understanding of the situation in Ethiopia, further explanation is required to make the results of this study useful information for other countries as well. | Indeed thank you so much for the valuable comment. We tried to revise the manuscript as much we can to make it clear to all the readers (please refer the manuscript with track changes for your quick reference). |
| Information such as the incidence rate of patients with drug-resistant infections and the proportion of drug-resistant bacteria in Ethiopia is not shown. Although the AMC in Ethiopia has been clarified, I would like you to discuss the relationship with drug-resistant bacteria. | Thank you for the valid comment. Now, we have included the same information in the discussion section (line number 295-300) of the revised manuscript. |
| I would like you to discuss the reasons for the increase in AMC in Ethiopia. You compare the reported values for Uganda and Tanzania, but readers will want to know the reasons for the change in AMC in Ethiopia. In particular, in many other countries, there are many reports of a decrease in AMC due to the impact of COVID-19. I would like you to discuss why such an impact was not seen. For example, is it possible that ordinary citizens used antibacterial drugs thinking they would also be effective against COVID-19? | Thank you for your concern and comments. However, despite the total AMC in DID is higher in Uganda (both before and during COVID-19) and Tanzania (before COVID-19) as compared to Ethiopia, there was still a decreased consumption in the Ugandan study, which might be partly because of the COVID-19 shutdown. We have also addressed this reason in the manuscript, particularly in the context of Ethiopia. |
| Can the general public purchase imported antibacterial drugs without a prescription? The routes by which antibacterial drugs are provided to the general public differ from country to country. Therefore, it would be more helpful if you explained in more detail the routes by which antibacterial drugs are obtained in Ethiopia. | Thank you for the comment. Dispensing or selling antimicrobials without prescription in Ethiopia is illegal or unethical by law. Regarding the routes of antimicrobial distribution, yes, now to make clear for readers, we included this information in the introduction section (Line 70-74). |
| What types of antibacterial drugs are not listed in the ATC classification and not included in this study? What proportion do they make up of the total? They need to be included in this study as supplementary materials or defined independently and included in AMC. | Thank you for the comment. Now, we included the same as a supplementary and indicated it as S1 file in the body of the Manuscript (Line 98-99). However, we can’t estimate what proportion they can represent since it is beyond the scope and WHO GLASS recommendation. |
| "The PLOS Data policy requires authors to make all data underlying the findings described in their manuscript fully available without restriction, with rare exception (please refer to the Data Availability Statement in the manuscript PDF file)." You have declared that you can use all the data, but you have not provided a link to where the data is stored. If it is difficult to post the raw data, I think you need to declare that correctly. | Thank you for the valuable comments and it is well taken. We will declare the same accordingly. |
| **Reviewer #4** |  |
| Page 3, Lines 65―66 Since previous studies did not aim to reveal antimicrobial consumption, I believe the phrase “failing to provide a complete picture of antimicrobial consumption across the country” is not an accurate expression. I recommend that the authors revise it to simply say, “these studies did not aim to reveal the complete antimicrobial consumption across the country.” | Indeed thank you so much for your valuable time and comments. This comment is taken as it is (lines 67-68). |
| Page 4, Lines 92―93 Data regarding drugs lacking ATC codes and DDDs seems important for improving antimicrobial usage. I recommend tabulating these drugs somewhere; the Supplementary Table would be a suitable option. | Thank you for the comment. Now, we included the same as a supplementary and indicated it as S1 file in the body of the Manuscript (Line 98-99). |
| Table 1 Please explain why D01BA was removed in the 2022 data and why A07AA was missing throughout the study duration. | The comment is valid and now to make it clear, we included in the revised manuscript Table 1, why D01BA (2022) and A07AA (entire study period) were not included in the report as a footnote (Line 144-146). |
| Table 2 Is it necessary to present antimicrobial volume in tons? Presenting data in tons is useful when human data are combined with animal data but may not be relevant when only human data is presented. | Thank you for the valid comment. Yes, you are right. WHO and others not considered tones as main metrics for AMC report. We included tones, as an additional information. |
| Table 6 We cannot calculate DID using the Ethiopian AWaRe category because the classification was not included in this manuscript. Is the Ethiopian AWaRe category necessary? I believe omitting the data categorized under the Ethiopian AWaRe framework will not negatively impact the manuscript. | Thank you for your concern. As per the comments provided by you and the editor, now we cited the Ethiopian AWaRe classification for those who are interested to see. Some of the antimicrobials categorized under access group and watch group were classified as watch and reserve group in the Ethiopian AWaRe classification, and vice versa. The Ethiopian AWaRe categorization only categorized antimicrobials included in the Ethiopian Essential medicine list which in were in total 33 (Access: 16, Watch: 10 and Reserve: 07). Whereas in the WHO AWaRe a total of (Access: 141; Watch: 87 and Reserve: 29). Example, Vancomycin was categorized as Reserve in Ethiopia while watch in WHO; Clarithromycin, Azithromycin and Norfloxacin are Watch in WHO and Access in Ethiopia. Erythromycin is not categorized in Ethiopian AWaRe, but in WHO it is Watch. Now the quick reference for the readers, we included the link (Line 134-136). |
| Page 6 The authors highlighted trends in antimalarial drug use, described them as “notable” in the text, and provided a figure. However, there is no discussion on antimalarials. Please elaborate on what is “notable” about these changes. | Thank for the valid comment and now revised the Table 2 description to be clear by including the antimalarial consumption issue as well (Line 162-164). |
| Discussion Antimicrobial consumption in Ethiopia was quite low, with the DID similar to that of developed countries. As DID in this report was calculated using import and production data, actual consumption may be even lower than the reported value. What reasons do the authors believe account for this difference compared to other African countries? | Thank you for the comments. The possible explanation might be that majority of our population live in the rural community (~85% of the population) where access to modern medicine is limited and medicine that is more traditional is practiced. However, this might be difficult for us to take it a standalone justification since we do not know example the Ugandan and Tanzania experience. |
| **Reviewer #6** |  |
| My suggestion: Kindly apply statistical analysis to your descriptive tables. Statistically compare data between 2020, 2021, and 2022 or some kind of Mann-Kendall trend test. Add this statistics to a column for each of the Table just before three year average. This will add context to your work, your as currently presented is inherently descriptive. | Thank you so much for the kind suggestion However, we run the Mann-Kendall trend test and found out that non-statistically significant difference across the years (p-value =0.083). In addition, we consulted literatures and statisticians; and recommended at least to have a sample of minimum 10 observations to run the Mann-Kendall trend test. As a result, we preferred not to present the findings in our manuscript. |
